# Children’s understanding of Aesop’s fables: relations to reading comprehension and theory of mind

**DOI:** 10.3389/fpsyg.2015.01448

**Published:** 2015-10-06

**Authors:** Janette Pelletier, Ruth Beatty

**Affiliations:** ^1^Dr. Eric Jackman Institute of Child Study, Ontario Institute for Studies in Education, University of TorontoToronto, ON, Canada; ^2^Department of Education, Lakehead UniversityThunder Bay, ON, Canada

**Keywords:** Aesop’s fables, reading comprehension, theory of mind

## Abstract

Two studies examined children’s developing understanding of Aesop’s fables in relation to reading comprehension and to theory of mind. Study 1 included 172 children from Junior Kindergarten through Grade 6 in a school-wide examination of the relation between reading comprehension skills and understanding of Aesop’s fables told orally. Study 2 examined the relation between theory of mind and fables understanding among 186 Junior (4-year-old) and Senior (5-year-old) Kindergarten children. Study 1 results showed a developmental progression in fables understanding with children’s responses becoming increasingly decontextualized as they were able to extract the life lesson. After general vocabulary, passage comprehension predicted fables understanding. Study 2 results showed a relation between young children’s theory of mind development and their understanding of fables. After general vocabulary, second-order theory of mind predicted children’s fables understanding. Findings point to the importance of developing mental state awareness in children’s ability to judge characters’ intentions and to understand the deeper message embedded in fables.

## Introduction

This paper describes a study of children’s developing understanding of story characters’ intentions and the resulting lesson that one can take away from Aesop’s well-known fables. The first study examines how fables understanding changes across grades from Junior Kindergarten (4 year-olds) to Grade 6 (12 year-olds), and the second study examines how “theory of mind” development in Kindergarten is related to fables understanding.

One of the goals of schooling is to bring children to an appreciation of stories: fantasy and fun, escapism, emotional arousal, food for thought, shared discussion and much more. However, many children are slow to or do not develop this appreciation. One reason may be that they do not develop the advanced comprehension skills to make insightful judgments about story characters’ mental states, in particular, their intentions and accompanying behavior. Understanding why this appreciation does not happen for some and how it does happen for others should be a concern for educators and researchers. We can begin by asking what reading – or listening to a story – entails. Both in reading and listening comprehension of narrative text, it requires the active construction of meaning by the reader based on a progressive understanding of story schema, and the ability to apply comprehension strategies including the identification of relations among characters, intentions, and actions based on information given in the text and prior knowledge. This requirement applies to younger children to whom a story is read, and to older children who are able to read for themselves. Indeed, it presents some interesting questions – when do young children begin to acquire an understanding of the intentions of the characters and to what extent do they understand the mental states of all the characters in order to fully comprehend the story?

Reading comprehension research has demonstrated that children develop a story schema through repeated exposure to stories (e.g., Anderson and Pearson, 1984; Paris and Paris, 2003). Most children’s stories employ a story structure consisting of a protagonist whose response to an initial event leads to an intention to achieve a goal and whose subsequent actions, relationships, conflicts, and resolutions result in a clearly stated outcome. It has been proposed that the ability to use this story structure schema is fundamental for children to identify, organize, and understand information from narrative text (Anderson and Pearson, 1984; Carrell, 1992; Baumann and Bergeron, 1993; Davis, 1994; Paris and Paris, 2003). Since children’s stories often center around a protagonist’s intention and the subsequent actions and relationships that develop in order that the intention be carried out, an understanding of characters’ mental states is needed for comprehending not only the sequence of events in a story, but also why the events took place and what judgments might be made about them. Indeed children’s storybooks are a ripe source of information for exposure to mental states (Cassidy et al., 1998; Dyer et al., 2000; Pelletier and Astington, 2004; Peskin and Astington, 2004). Nevertheless, true mental state understanding requires more than simple exposure to mental state terms (Peskin and Astington, 2004); it also requires personal, social, experiential, and linguistic interpretation (Astington, 1996; Nelson et al., 1998).

Several studies assessing students’ retellings of short stories have shown that children in the primary grades tend to recount stories as a list of actions (Carnine et al., 1982; McConaughy et al., 1984). A study by Nezworski et al. (1982) concluded that children in Kindergarten and Grade 3 prefer to tell stories as a series of factual events, rather than to describe “uncertain internal states.” McConaughy et al. (1984) found that children are less likely than adults to mention character intentions when recalling a story and claim that children and adults use different story schemata. Children tend to focus on what happened by listing a series of actions that took place, and adults focus on why things happened including statements about characters’ thoughts, beliefs and intentions. Other research has shown that young children are able to integrate plot actions with characters’ thoughts and beliefs, but only if they have the mental state understanding and mental state language that allow them to link action with consciousness (Pelletier and Astington, 2004). When the main character’s intention is explicitly articulated, children from Kindergarten to Grade 6 are able to identify the mental state and related goal of the protagonist but the stories still tend to be retold as a sequence of events rather than as a holistic narrative of interrelationships and goal achievement (Feathers, 2002). Nelson has maintained that while children may hear and use mental state language, it is their increasing experience in the social world that allows them to fully understand the meaning of the term and to give a mentalistic interpretation to behavior (Nelson et al., 1998).

Bruner (1986) described the comprehension of stories as existing on two planes. One is understanding a structure in which characters play out a sequence of events in order to reach a conclusion; this is the action plane. The other is understanding that characters are goal-driven based on intention and act because of their thoughts and beliefs that may or may not be accurate; this is the plane of consciousness (see also Pelletier and Astington, 2004). Fables are a particular kind of story with their own structure and lesson to be learned. To understand fables, it is necessary to understand the story on both of Bruner’s levels. Fables are an effective way to assess children’s comprehension of character intentions because the positive or negative attributes of a character do not always correspond to that character’s outcome. In many Aesop’s Fables, the lesson is not that the “bad character” is punished and the “good character” is rewarded. Rather, they are cautionary tales in which (typically) an animal symbolizing a human character flaw (greed, arrogance, stupidity, timidity, naïveté, carelessness) is deceived by another character, inadvertently helping the deceiving character to fulfill his/her intention. In many cases the “good character” can be weak and the “bad character” strong. This kind of characterization sends the message that power and evil can win out over innocence and good will unless thoughtful action is taken (Clayton, 2008). Alternatively, fables may exploit a situation in which a good character attribute is revealed (kindness, empathy, intelligence), resulting in an unexpected positive outcome. An essential component of comprehending the meaning of fables is knowing the relationship of who is tricking or surprising whom, and for what purpose. To that end, children’s understanding of mental states may be needed for competence in narrative structure particularly when it involves deception (Sodian et al., 1991; Gamannossi and Pinto, 2014), as is common in Aesop’s fables. Second order theory of mind, in particular, has been linked to understanding of deception (Wimmer and Perner, 1983) because it involves beliefs about another’s beliefs or intentions.

Fables were used in a study by Shannon et al. (1988), one of the first specifically designed to assess children’s comprehension of character intentions. Children and adults listened to or read fables in which character intentions were implicitly or explicitly stated. The results indicated that children found it more difficult than adults to identify and articulate character intentions, even when directly questioned about stories in which the character intentions were explicitly expressed. The authors’ conclusion was that elementary students may not automatically put themselves in the place of main characters and try to solve the problems they face, but rather may rely on a comprehension strategy that focuses on characters’ actions as opposed to goals or intentions.

Fables are didactic stories that were initially composed and orally transmitted in order to teach a pertinent life lesson and to guide people in how to live a morally upstanding life (Tomasulo and Pawelski, 2012). A structural understanding of the plot must be accompanied by an ability to infer the overarching moral that the author is attempting to convey (either implicitly embedded within the text, and/or explicitly stated at the end). When the moral is not explicitly stated, the reader must consider the outcome of the fable in relation to the intended actions of the characters, and in relation to their understanding of fair and equitable consequences (Dorfman and Brewer, 1994). As in the research on story understanding and retelling, there are developmental differences in fables understanding. Children in Grade 5 comprehend fables better than their younger Grade 3 peers, but not as well as college students (Narvaez et al., 1998). Dorfman and Brewer (1994) showed that adults find stories that have moral outcomes more meaningful and comprehensible than similar stories that do not have a moral outcome.

Interestingly, Aesop’s fables feature animals as protagonists rather than humans. In some contexts, the use of non-humans is meant to illuminate the human experience through “humans in disguise” (Sutton-Spence and Napoli, 2010, p. 442) in a metaphoric way. This kind of anthropomorphism is ubiquitous across cultures (Tehrani, 2013) and is considered acceptable for evoking emotion and appealing to broad audiences (Sutton-Spence and Napoli, 2010). However, behavioral scientists point out some potential drawbacks of anthropomorphism when it concerns teaching children scientific understanding of the natural world (Ganea et al., 2014). Yet Shettleworth (2012), using training tasks inspired by Aesop’s fables (for example, the thirsty stork using stones to raise the water level in a pitcher), along with neuroscientific imaging, maintains that animals can indeed show insight. Clayton (2008) argues that anthropomorphism illustrates that hierarchies in the animal world are analogous to human hierarchies in the context of everyday life. There must be similarities between animal and human behavior, otherwise “the animal fable would not exist” (Clayton, 2008, p. 183). Although animals can get themselves into similar situations as humans, all would agree that animals can not use reason to solve a problem. Thus the anthropomorphized life lesson is useful in showing human audiences that they have the advantage of mentalistic reasoning to help them avoid or escape the unfortunate situation.

An understanding of fables requires that the individual take the perspective of the main characters, with an appreciation not only of who is tricking whom, but also with an educational goal of teaching children a life lesson. To date, most studies have compared the poorer understanding of young children to the superior understanding of adults. They have demonstrated that an understanding of both characters’ and author’s intentions increases the ability to understand the meaning of the fable. Studies have not, however, specifically described the development of the underlying cognitive processes related to intention in fables comprehension.

One theory related to cognitive processes in story comprehension is that children need to be able to conceptualize and construct internal mental states (Bruner, 1986; Pelletier and Astington, 2004; Peskin and Astington, 2004). In order to understand character intentions, children need to be able to conceive that individuals (in stories and in reality) behave in ways that will result in their attainment of a goal, and that behavior is driven by internal mental states such as desire (Gamannossi and Pinto, 2014). Fables add another dimension – the complexity of deception or surprise. In most fables, both of the characters desire something, but one character formulates a deception or surprise based on the desires of the other character and so attains his goal. The reader must simultaneously understand the internal mental states of both characters, and understand that for one character the representation of reality may be inaccurate and thus offer opportunities of which the other character can take advantage. For instance, consider Aesop’s story of the fox and the crow, a story that involves deception:

*A big black Crow was sitting on a branch of a tree with a piece of cheese in her beak when she was seen by a hungry Fox. The Fox walked under the branch, looked up at the Crow, and said, “What a noble bird you are! Your beauty is without equal and the color of your feathers is exquisite. If your voice is as sweet as your looks, then I think you are the Queen of the Birds.” The Crow was very flattered by the Fox’s compliments and, just to show him that she could sing, she opened her mouth to caw. But as soon as she opened her mouth, the cheese fell to the ground, where it was snatched up by the clever Fox (Hague, 1985)*.

In this story, the reader or listener must understand the mental states of each of the characters – the crow’s vanity, the fox’s hunger, the fox’s recognition of the crow’s vanity, and the crow’s mistaken belief about the fox’s opinion of her.

As in a study that employed folktales and fairytales (Ratner and Olver, 1998), it was concluded that children’s increasing ability to understand and appreciate fables may be linked to their own growing awareness that there is a difference between reality and the internal representations that each individual has to represent reality, and that these internal representations or beliefs may be false.

### Links to Theory of Mind

Numerous studies have described a shift in children’s cognitive ability to conceptualize an internal state of mind; typically the development of the “false belief” takes place at about 4 years of age (Astington, 1990, 1993; Wellman et al., 2001). Researchers argue that this shift in understanding constructed by the child – albeit developmentally constrained – develops over the course of the preschool years, and is characterized differently at different ages. That is, at approximately 4 years of age, children come to acquire a “first order” theory of mind understanding (he thinks that × is in the box), whereas between approximately 5 and 6 years of age children come to acquire a “second order” theory of mind (he thinks that she thinks that x is in the box; Wimmer and Perner, 1983; Astington et al., 2002). Theory of mind understanding becomes more recursive as children get older, for example enabling young adolescents to develop a greater understanding of self (Bosacki, 2000). Further, language makes significant contributions to theory of mind understanding and this relationship is interconnected implicitly and explicitly across ages and language group status (Antonietti et al., 2006; San Juan and Astington, 2012; Pelletier et al., 2014).

The fables task used in this study was designed to ascertain whether children’s increasing ability to identify characters’ intentions is related to their developing theory of mind. In the present study, both first order and second order theory of mind tasks were used, as most of the children were 4 years-old and older. Furthermore, second order theory of mind tasks (but not first order) have been shown to predict children’s performance in reading comprehension tasks among second language learners (Pelletier, 2006) and in cause-evidence distinction tasks (Astington et al., 2002). Cause-evidence tasks tap children’s emerging understanding of why an event happened (cause) in contrast to how they know an event happened (evidence). Younger children confuse cause and evidence; for example, “I know the floor is wet because John spilled water” (cause) and “I know the floor is wet because I stepped in it” (evidence).

Given the comprehensive body of research showing that children’s theory of mind develops rapidly during the preschool and early school years, we hypothesized that children’s understanding of fables would increase from Kindergarten to Grade 6, and that a large incremental shift in understanding would take place between ages 4 and 6 years when children begin to acquire a second order theory of mind understanding. We thought that children’s ability to understand fables would be related to general reading comprehension; that is, children who are able to extract the “lesson” of the fables as evidenced by increasingly decontextualized responses, would also perform well on standardized reading comprehension tasks. Typically these standardized tasks measure basic level comprehension skills but do not measure intentional understanding. The fables task goes further in assessing comprehension by linking text comprehension with an understanding of underlying intentions and relatedly, the moral of the story. We further hypothesized that children’s developing ability to understand epistemic states as measured by theory of mind tasks at 4 and 5 years of age would predict their ability to understand the intentional states of all characters in the fables task and to articulate the moral of the story based. Thus two separate but related phenomena were being examined: (1) grade level developmental progression of children’s understanding of character intentions in children’s understanding of fables and its links to general reading, and (2) the relation between children’s theory of mind and their understanding of fables between 4 and 5 years of age.

## Materials and Methods

Data from two separate studies were used in the analysis. Study 1 data provide information on grade level and ability group differences in fables task performance and the relation to reading ability. Study 2 data provide information on the relation between fables task performance and theory of mind understanding. Both studies were approved by two research ethics boards; Study 1 ethics boards included the University of Toronto and the Institute of Child Study Research Ethics Committees. Study 2 ethics boards included the University of Toronto and the two school board (public and Catholic) external research committees in the Region of Peel, to the west of Toronto.

### Participants

Participants in Study 1 include 172 children from Junior Kindergarten (JK- 4-year-olds) and Senior Kindergarten (SK- 5-year-olds) to Grade 6 (12-year-olds) in a private university laboratory school in Toronto, ON, Canada. All children in these grades participated in the study except in cases in which a child moved in or out of the school during the study’s duration. Each class had either 21 or 22 children. There were 87 girls and 85 boys. The school population includes approximately 30% from visible minority and lower socioeconomic groups; however, most children are from middle-income families. Due to the generally high academic performance of children at this school, results may reflect higher achievement levels than in public schools in the Toronto area. However, results are useful as indicators of developmental differences.

Participants in Study 2 include 186 children in Junior (4-year-old) and Senior Kindergarten (5-year-old) classes from 5 public schools in the Greater Toronto area. More than 60% of the children spoke English as an additional language and represented a wide range of cultural, racial, linguistic, and socioeconomic diversity. Close to 40% spoke English as a first language. Second language groups primarily include Hindi, Gujarati, Punjabi, Urdu, Tamil, Vietnamese, and Chinese. Children who were judged by their teachers as not able to understand English well enough to fully respond to the questions were not included. There were 101 girls and 85 boys in Study 2; 113 were in Junior Kindergarten and 83 were in Senior Kindergarten. The analyses were carried out with participants for whom complete data were available.

### Procedures for Both Studies

All participating children were withdrawn individually from their classrooms to a nearby familiar area and were administered the battery of measures by a trained graduate student teacher candidate. Testing time ranged according to grade level but averaged approximately 40 min per child. Children were not made to participate if they were shy, unwilling or tired. All tasks were administered in counterbalanced fashion by the use of two lists. Theory of mind tasks in Study 2 were also counterbalanced for order of administration of the individual task items. In past research (e.g., Astington et al., 2002) and in the present study, there were no effects of task administration order.

### Measures

#### Vocabulary

The Peabody Picture Vocabulary Test III Revised (Dunn and Dunn, 1997) was administered to all participants following standardized procedures. Raw scores were used and age controlled in the analyses. This measure was given to all children in both studies.

#### Reading

Two standardized measures of reading were employed to address developmental differences. Standardized procedures were followed. For older children (Grades 2–6), three subtests of the Woodcock Reading Mastery Test (WRMT; Woodcock, 1998) were used in Study 1: Passage Comprehension, Word Attack and Word Identification. These were chosen because they include basic skills (Word Attack and Word Identification) as well as understanding (Passage Comprehension). For younger children in Grades Junior Kindergarten – Grade 1 in Study 1, for whom the WRMT was too difficult, the Test of Early Reading Ability-III (Reid et al., 2001) was employed. Because all children in Study 2 were in kindergarten, the TERA was administered to all children in Study 2. The TERA includes three subtests: Alphabet Knowledge, Conventions of Print, and Meaning. The first two subtests measure basic early reading skills and the Meaning subtest measures understanding. Raw scores representing the total of the three subtests of the TERA were used in the analyses.

#### Teacher Ratings of Reading

Each classroom teacher in Study 1 rated each child in reading ability by group: low, medium or high. Teachers’ ratings were global subjective measures of reading based on children’s skills and comprehension and that teachers felt may have captured a broader picture beyond the standardized tests. Teachers were collaborators in this research and were interested in the relation among the measured skills and comprehension and their own global impressions of children’s understanding.

#### Fables Comprehension

An experimental measure that assesses children’s basic story comprehension as well as deeper comprehension of character motivations and the moral of the story was administered to all participating children in both studies. Two Aesop’s fables tasks were used (The Fox and the Crow, The Fox and the Goat; fables used from Hague, 1985); for all children and for each task a fable was read by a researcher while the child was shown an accompanying illustration. The child was then asked three basic knowledge and comprehension questions (Questions 1–3), coded as correct/incorrect (0 or 1) and one lesson/moral of the story question (Question 4), coded on a scale from 0 to 5. Examples from the Fox and the Crow are:

Q1 : What did the fox see up in the tree branch?
Q2 : Why did the crow open her mouth to sing?
Q3 : Is someone playing a trick? Who?
Q4 : What is the moral/lesson of this story? (probe: What can you learn from this story?)

The maximum raw score for both fables was 16. The maximum raw score for Question 4 (moral question) was 10 (two fables × maximum five points). Coding for the final question measured children’s fable comprehension from recognizing the character’s intention to “trick” through to an ability to extract the “life lesson” from the fable in an increasingly decontextualized fashion. Younger children tend to respond within the context of the story (“the lesson is that you should not listen to foxes”) whereas older children tend to respond in a more decontextualized fashion (“the lesson is that you should not listen to flatterers”). Coders were trained together until they reached consensus on 100% of trial codes. Ten percent of children’s responses in the dataset were then double-coded for reliability purposes and agreement reached 90% as reported in previous studies using this method of training (e.g., Timmons et al., 2015). Any differences in coding tended to be in the distinction between scores 4 and 5. A series of acceptable responses was then developed for a score of 5 (a standard cliché) to reduce this error. The procedure for Question 4 was as follows:

0 = incorrect story fact, nonsense
1 = correct story fact (the fox asked the crow to sing)
2 = reference to trick (the fox wanted to trick the crow)
3 = reference to lesson tied directly to story context (we can learn not to sing just because a fox asks us to do that)
4 = reference to lesson decontextualized beyond the story (we can learn not to show off because then we might lose something)
5 = broad life lesson/cliché (do not trust flatterers/people who just say nice things to get something)

#### Theory of Mind

Two batteries of theory of mind tasks consisting of a first order and a second order false belief were used with children only in Study 2. Both tasks have been employed in traditional theory of mind studies (e.g., Wimmer and Perner, 1983; Astington and Jenkins, 1999; Astington et al., 2002). The first order false belief task measures children’s understanding of a character’s mental state (e.g., “he knows that…”), whereas the second order false belief task measures children’s understanding of an embedded mental state proposition (e.g., “she knows that he knows that…”). Children were given four first order stories for a possible total of 10 correct points and if they passed, were given 2 second order stories for a maximum total of 16 points (justification responses are scored from 0 to 4). A more detailed copy of the scoring system may be obtained from the first author.

In summary, Study 1 allowed an investigation of grade level differences in fables understanding and its relation to a number of standardized reading assessment tools appropriate for a range of grade levels. Study 2 allowed an investigation of fables understanding at one time point, in Kindergarten, in relation to theory of mind and early reading ability.

## Results Study 1

The following are representative grade-level examples of children’s responses in the Fox and the Crow example (the crow who drops the cheese in response to the fox’s flattery) to Question 4, “what can you learn from this story?”

JK – “About being a queen”; About foxes
SK – “Not to listen to a fox”; Foxes are mean
Gr1 – “Not to open your mouth when you have something in your mouth to show that you can do it”; Do not just do things when a fox says so
Gr2 – “Not to fall for that kind of stuff”; Do not show off
Gr3 – “Do not be tricked”; Do not think you’re such a beautiful bird
Gr4 – “Think before you do something”; Be wary of people who ask for things
Gr5 – “Do not listen to strangers that are really smart”; Be careful of people who always say nice things to you
Gr6 – “Sometimes people flatter to get what they want”: Beware of flatterers.

The means, minimum scores, maximum scores and standard deviations are presented in **Table 1**.

**Table 1:** Means, ranges, and standard deviations across grades in fables task performance (maximum possible score = 16 for both fables).

	JK	SK	Gr 1	Gr 2	Gr 3	Gr 4	Gr 5	Gr 6
	*N* = 22	*N* = 21	*N* = 22	*N* = 21	*N* = 21	*N* = 21	*N* = 21	*N* = 22
Mean	4.73	7.95	9.45	10.10	10.38	10.57	10.81	12.91
Minimum	2.00	1.00	4.00	5.00	5.00	7.00	6.00	8.00
Maximum	9.00	12.00	14.00	16.00	14.00	15.00	15.00	16.00
*SD*	1.75	3.04	3.17	3.51	2.62	2.56	2.36	2.51

*JK, Junior Kindergarten (4 years), SK, Senior Kindergarten (5 years).*

**FIGURE 1:**
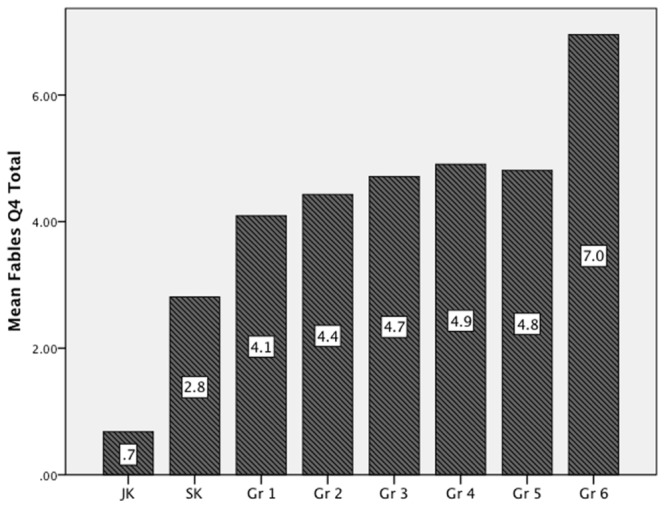
**Grade level differences in responses to the moral question**.

A One-Way ANOVA was used to examine grade level differences in children’s fables task performance for Question 4 alone (the moral/lesson question). Mean scores ranged between.68 (JK) and 7.0 (Gr. 6) (see **Figure 1**). Findings represent a significant difference by age [*F*(7,163) = 11.8, *p* < 0.001]. A Bonferroni *post hoc* comparison showed the greatest leaps were initially between JK-Grade 1 and again at Grade 6. A Bonferroni correction was not used as Sedgwick (2012) suggests that this test may be overly conservative and can prevent the identification of significant findings when many comparisons are made, in this case grade levels.

**FIGURE 2:**
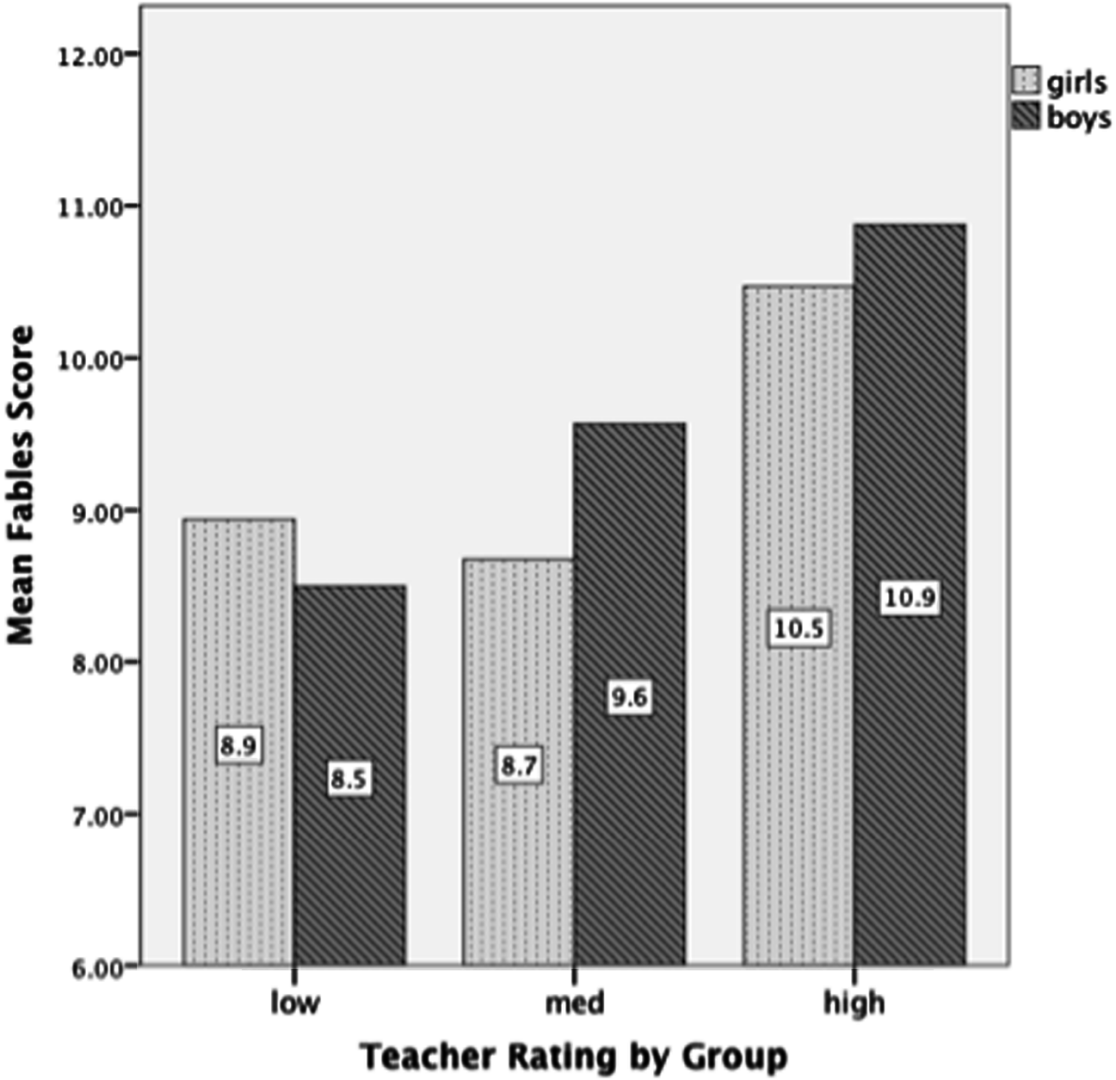
**Mean fables total scores for low, medium, and high reading groups**.

The next analysis compared children’s fables task performance in relation to teacher reports of whether children were in the high, medium, or low reading groups in the class. Results showed significant differences between teacher rating groups, that is, children in the highest teacher-rated reading groups were those who received the highest scores on the fables task [*F*(2,168) = 5.32, *p* < 0.01; See **Figure 2**]. There were no gender differences.

In order to examine the interrelations among the variables, a partial correlational analysis controlling for age was carried out. It should be noted that two reading measures needed to be used to address developmental differences, the TERA for younger children and the WRMT for older children; thus reading results are reported separately for age groups. The fables task was modestly but significantly correlated with vocabulary [*R*(62) = 0.35, *p* < 0.01, for younger children; *R*(85) = 0.41, *p* < 0.001 for older children] and with the passage comprehension subtest [*R*(88) = 0.38, *p* < 0.05] but less with word identification and word attack skills. Teacher ratings of high, medium or low reading performance were significantly correlated with vocabulary [*R*(65) = 0.29, *p* < 0.05 for younger children; *R*(85) = 0.34, *p* < 0.001 for older children], with TERA performance for the younger children [*R*(65) = 0.36, *p* < 0.05], passage comprehension for the older children [*R*(88) = 0.51, *p* < 0.001], word identification [*R*(88) = 0.27, *p* < 0.05] and word attack [*R*(88) = 0.30, *p* < 0.01], and with fables task performance for all children [*R*(168) = 0.24, *p* < 0.005].

A stepwise regression analysis was used to test the relative contributions of the reading and vocabulary measures to fables task performance. In the first regression, vocabulary, passage comprehension, word identification, and word attack skills were entered. Only vocabulary made an independent contribution to fables task performance (*R* = 0.43, *R* Square = 0.19, *F* = 13.24, *p* < 0.001). In a second regression analysis, vocabulary was not entered. This time, only passage comprehension made a significant contribution to fables task performance (*R* = 0.42, *R* Square = 0.18, *F* = 12.2, *p* < 0.001).

## Results Study 2

The next set of results draws from the data in Study 2. The first analysis presents means and standard deviations for Junior Kindergarten and Senior Kindergarten children on the dependent variables of vocabulary, early reading, theory of mind, and fables task performance. First order theory of mind was administered to all children and second order theory of mind was administered to children who correctly answered all items on the first order task (See **Table 2**).

**Table 2:** Means and Standard Deviations for JK (Junior Kindergarten) and SK (Senior Kindergarten) children.

JK or SK		PPVT	TERA	1st order	2nd order	Fables
JK	Mean	48.19	12.17	6.29	6.43	3.94
	*N*	108	109	103	35	96
	*SD*	22.18	8.01	2.42	2.44	2.37
SK	Mean	63.56	21.25	7.40	7.05	5.42
	*N*	79	79	83	43	79
	*SD*	22.91	9.85	2.33	2.21	2.69

For all variables, the differences between Junior Kindergarten (JK) and Senior Kindergarten (SK) performance were significant at the.001 level, except for first order Theory of Mind which was significant at the 0.05 level and for second order Theory of Mind which was not significant.

The next analysis examined correlations among the variables partialling out age. Second order, but not first order theory of mind was significantly correlated with fables task performance (*r* = 0.40, *p* < 0.001). Vocabulary was likewise correlated with fables task performance (*r* = 0.57, *p* < 0.001) and with second order theory of mind (*r* = 0.33, *p* < 0.01), but not with first order theory of mind, and only modestly with early reading ability as measured by the TERA (*r* = 0.26, *p* < 0.05; see **Table 3**).

**Table 3:** Partial correlations controlling for age.

	PPVT	TERA	1st order	2nd order	Fables
PPVTRAW		0.26^∗^	0.22	0.33^∗∗^	0.57^∗∗∗∗^
TERA			0.21	0.38^∗∗∗^	0.27^∗^
1st order				0.19	0.23
2nd order					0.40^∗∗∗^

*^∗^p < 0.05, ^∗∗^p < 0.01, ^∗∗∗^p < 0.005, ^∗∗∗∗^p < 0.001.*

Finally a stepwise regression analysis on the dependent variable of fables understanding (fables total score) with the factors of age, vocabulary, early reading ability, first order theory of mind, and second order theory of mind, showed that vocabulary and second order theory of mind contributed to children’s understanding of fables. Vocabulary accounted for 36% of the variance [*R* = 0.60, *R* Square = 0.36, *F*(1,67) = 38.1, *p* < 0.001] and second order theory of mind accounted for an additional 6% of the unique variance [*R* = 0.64, *R* Square = 0.42, *F*(2.66) = 22.7, *p* < 0.05].

## Discussion

Taken together, the results show that as children age, they gain an increasing understanding of fables. Consistent with the first hypothesis, as children became older their comprehension of text became increasingly decontextualized; that is, their understanding progressed from identifying story facts to extracting a life lesson that was less explicitly tied to the story action and was more implicitly tied to the mental states or intentions of the story characters. In the example of the Fox and the Crow, 4- and 5-year-old children in Study 1 were more likely to think about the Crow as becoming a queen or the Fox as the “bad guy.” However, 11- and 12-year-old children in Grades 5 and 6 were more likely to think about a larger life lesson, in this case the dangers of succumbing to flattery. This developing awareness is likewise related to general reading comprehension as measured by standardized passage comprehension tests and to general vocabulary knowledge. Vocabulary knowledge is often used as a proxy for general intelligence and thus it is not surprising to find that only vocabulary predicted children’s performance on the fables task; passage comprehension, word identification and word attack skills did not predict. However, when the regression analyses were carried out without entering vocabulary, children’s performance in passage comprehension predicted performance on the fables task but other reading skills did not. Interestingly teacher ratings of children’s reading ability also correlated with vocabulary and fables task performance but not to discrete skills of word identification and word attack. It is important to note that there was very little variability in the standard vocabulary scores of the Study 1 children. Standard scores were calculated for Study 1 children and all classes were in the above to well above average range. While this fact may reduce the generalizability of the age-related stages, it also reduces error due to variability in verbal intelligence. The age-related stages may simply apply to children in the next grade in other school systems. There were no gender differences in this study despite research showing young girls’ higher verbal performance in narrative tasks (e.g., Fivush and Zaman, 2015). However, comprehension of mental state stories, including false belief tasks, may differ from production of mental state narratives. For example, Charman et al. (2002) reported a very slight advantage in false belief task performance for girls which did not hold over time. Thus it is not surprising that gender differences in mental state story comprehension were not found in the present study.

In Study 2, there were also significant differences between Junior and Senior Kindergarten in all measures including first order theory of mind but with the exception of second order theory of mind which fewer kindergarten children (78 rather than 187) were administered due to the level of difficulty. Applying these findings to individual differences among children in classrooms – without an ability to move beyond earlier stages in a taxonomy in which reading moves from simple knowledge of facts to ability to synthesize, apply and judge (e.g., Bloom, 1956), children remain at lower levels of reading comprehension, only having basic knowledge of the facts without understanding the characters’ intentions and larger moral “purpose” of the tale. They lack an ability to synthesize what they read, to apply it to their own and other life experiences, and to think critically about the message of the text. Fables comprehension, while linked to story comprehension, also requires an ability to understand intention. Extracting and articulating a decontextualized life lesson is dependent upon understanding the character intentions that hold the story together. The fables task is thus useful for quickly measuring children’s ability to think critically about the intentions of the characters and the overall message of the story. It offers intriguing insights as to how children think about the deeper message of the story. A limitation of Study 1 was the lack of theory of mind data; Study 2 addressed this issue.

The second hypothesis, that Kindergarten children’s performance on the fables task would be tied to theory of mind development, was supported in Study 2. In fact, theory of mind understanding contributed most to Kindergarten children’s understanding of the fables beyond the contribution of general vocabulary. This is in contrast to research that showed no unique contribution of theory of mind to story comprehension (Strasser and del Río, 2013). In that study vocabulary mediated the effect of executive function in predicting story comprehension. In our study, executive function skills were not included; thus our results should be interpreted cautiously given the lack of executive function data. There are several other points to make here. The first is that more than 60% of the children in Study 2 spoke a language other than English as their first language. Although all children who participated in the study were deemed by their teachers to be competent enough in English to participate, their scores on the standardized vocabulary test were lower than for children who spoke English as a first language. Thus it may not be surprising that vocabulary made less contribution to fables understanding in Study 2 than in Study 1. Another interesting point that has support in previous research (Astington et al., 2002) concerns the greater predictive power of the second order theory of mind tasks than of the first order tasks. Second order theory of mind tasks require children to be aware of the mental states of both characters and to be able to make inferences and judgments about the characters’ intentions based on their mental representations. Similarly, fables understanding requires children to keep in mind the mental states of all the characters in order to understand who is tricking whom (if applicable) and to understand the characters’ intentions. It is not surprising therefore, that second order theory of mind is important to fables understanding.

In summary, this study has described the relation between fables understanding and story comprehension and has suggested the process by which fables understanding and theory of mind development may be linked. These relations provide useful information for educators: the importance of mental state understanding which may come about through explicit talk about mental states in the classroom, and the value of “getting inside children’s minds” to assess the extent to which they truly understand the meaning of what they read and hear in stories.

## Conflict of Interest Statement

The authors declare that the research was conducted in the absence of any commercial or financial relationships that could be construed as a potential conflict of interest.
